# Impact of acute kidney injury on graft outcomes of deceased donor kidney transplantation: A nationwide registry-based matched cohort study in Korea

**DOI:** 10.1371/journal.pone.0260076

**Published:** 2021-11-17

**Authors:** Jane Ha, Cheol Woong Jung, Sunkyu Choi, Myung-Gyu Kim, Jun Gyo Gwon, Joong Kyung Kim, Chan-Duck Kim, Ji Won Min, Jaeseok Yang, Curie Ahn, on behalf of the Korean Organ Transplantation Registry Study group

**Affiliations:** 1 Department of Medicine, Korea University College of Medicine, Seoul, Korea; 2 Department of Surgery, Korea University Anam Hospital, Seoul, Korea; 3 Department of Biostatistics, Korea University College of Medicine, Seoul, Korea; 4 Department of Internal Medicine, Korea University Anam Hospital, Seoul, Korea; 5 Department of Internal Medicine, Bongseng Memorial Hospital, Busan, Korea; 6 Department of Internal Medicine, School of Medicine, Kyungpook National University Hospital, Daegu, Korea; 7 Division of Nephrology, Department of Internal Medicine, Bucheon St. Mary’s Hospital, Bucheon, Korea; 8 Department of Nephrology, Seoul National University Hospital, Seoul, Korea; Istituto Di Ricerche Farmacologiche Mario Negri, ITALY

## Abstract

**Background:**

Favorable long-term and short-term graft survival and patient survival after kidney transplantation (KT) from deceased donors with acute kidney injury (AKI) have been reported. However, few studies have evaluated effects of donor AKI status on graft outcomes after KT in Asian population. Thus, the purpose of this study was to evaluate graft function after KTs from donors with AKI compared to matched KTs from donors without AKI using a multicenter cohort in Korea.

**Methods:**

We analyzed a total of 1,466 KTs collected in Korean Organ Transplant Registry between April 2014 and December 2017. KTs from AKI donors (defined as donors with serum creatinine level ≥ 2 mg/dL) and non-AKI donors (275 cases for each group) were enrolled using a 1:1 propensity score matching. Graft outcomes including graft and patient survival, delayed graft function (DGF), rejection rate, and serially measured estimated glomerular filtration rate (eGFR) were evaluated.

**Results:**

After propensity matching, KTs from AKI donors showed higher rate of DGF (44.7% vs. 24.0%, *p* < 0.001). However, the rejection rate was not significantly different between the two groups (KTs from AKI donors vs. KTs from non-AKI donors). eGFRs measured after 6 months, 1 year, 2 years and 3 years were not significantly different by donor AKI status. With median follow-up duration of 3.52 years, cox proportional hazards models revealed hazard ratio of 0.973 (95% confidence interval [CI], 0.584 to 1.621), 1.004 (95% CI, 0.491 to 2.054) and 0.808 (95% confidence interval [CI], 0.426 to 1.532) for overall graft failure, death-censored graft failure and patient mortality, respectively, in KTs from AKI donors compared to KTs from non-AKI donors as a reference.

**Conclusions:**

KTs from AKI donors showed comparable outcomes to KTs from non-AKI donors, despite a higher incidence of DGF. Results of this study supports the validity of using kidneys from deceased AKI donors in Asian population.

## Introduction

Kidney transplantation (KT) is the treatment of choice for eligible patients with end-stage renal disease (ESRD) which is superior to any other treatment modalities including renal replacement therapies [1, 2]. However, the issue of organ shortage has been raised because the incidence of ESRD is increasing while the donor pool remains relatively unchanged. There have been efforts to maximize the utilization of donated kidneys. Formalized definition and use of expanded criteria donor (ECD) donor [3] have allowed more patients to benefit from KT.

Kidney discard rate from donors with acute kidney injury (AKI) is significantly higher than that from donors without AKI [4, 5] because serum creatinine level of donor has been recognized as one of critical factors contributing to poor outcomes after KT. However, accumulating clinical evidence including long-term observations supports comparable patient survival and graft survival of KT from donors with and without AKI [5–12]. Main concerns about using kidneys from donors with AKI have been primary non function [13]. However, many of previous reports had a single-center design [7, 12, 14–18]. There have not been sufficient studies for supporting the safety and efficacy of KTs from donors with AKI in Asian population [9, 14].

Therefore, we conducted a matched cohort study using a nationwide multicenter cohort in Korea to evaluate effects of deceased donor AKI status on graft function after KT presented by estimated glomerular filtration rate (eGFR) with minimized selection bias, and to assess adequacy of KT from donor with AKI in Asian population.

## Patients and methods

### Study population

The Korean Organ Transplant Registry (KOTRY) is a web-based national transplant registry established in 2014. It includes demographic and clinical data of donors and recipients from 32 centers. The database was accessed on January 2020 for data collection, and all cases of KT from deceased donors and registered in KOTRY between April 2014 and December 2017 were eligible for this study. Exclusion criteria were en-bloc or dual KTs, KTs from donor aged less than 19 years, and cases with missing variables including donor serum creatine, height, weight, presence of hypertension, or presence of diabetes. The KOTRY study was reviewed and approved by the Institutional Review Board of Korea University Anam Hospital (approval number: 2014-0272-024). All data were fully anonymized before researchers accessed the database. The Institutional Review Board waived the requirement for informed consent. None of the transplant donors in the dataset was from a vulnerable population and all donors or next of kin provided written informed consent that was freely given. This study followed the Strengthening the Reporting of Observational Studies in Epidemiology reporting guidelines.

### Data collection

Primary data included information of donors, information of recipients, and transplantation-related factors. Donor factors included age, height, weight, body mass index (BMI), cause of death, serum creatinine, presence of diabetes or hypertension, smoking status, and human leukocyte antigen (HLA) types (HLA-A, -B, -DRB1). Kidney donor profile index (KDPI) and kidney donor risk index (KDRI) were calculated based on donor characteristics [19]. Recipient factors included primary cause of renal disease (diabetes, hypertension, glomerular disease, tubular interstitial disease, polycystic kidney disease, or others), history of kidney transplantation, presence of diabetes or hypertension, age, height, weight, BMI, and HLA types. Transplant-related factors included cold ischemia time (CIT), results of donor specific antigen (DSA) test, agents of induction therapy, and maintenance immunosuppressants. HLA mismatch score was calculated by counting the number of HLA matched loci of HLA-A, -B, and -DRB1 out of six. Clinical outcomes included serum creatinine levels of recipients measured at 6 months, 1 year, 2 years, and 3 years after KT, delayed graft function (DGF, defined as the need for renal replacement therapy during the first week after KT), primary nonfunction (PNF), graft loss, patient loss, biopsy proven rejections within 1-year post-KT (borderline rejections, T-cell mediated, antibody mediated, and mixed rejections), and BK nephropathy. eGFR in each follow-up period was calculated using demographic factors and serum creatinine level using the Modification of Diet in Renal Disease study equation [20].

### Statistical analysis

Continuous variables and categorical variables are presented as means with standard deviations and frequency with percentages, respectively. AKI donor was defined as donor with terminal serum creatinine level (the last serum creatinine level measured before KT) ≥ 2.0 mg/dL. We used a 1 to 1 propensity score matching from a logistic regression to minimize the difference in baseline covariates between AKI donors and non-AKI donors. We performed propensity score matching in consideration of donor age, sex height, weight, presence of hypertension and diabetes, and cause of death using the caliper matching. To assess the balance of variables used in the matching, standardized mean differences (SMDs) were checked.

Differences in continuous outcomes and categorical outcomes between the two matched groups were investigated using independent t-test and chi-squared test, respectively. A generalized estimating equation model was used to estimate statistical significance of variance in eGFR between groups. Bonferroni correction was used for comparing eGFR at specific time point. Graft failure was defined as a return to dialysis or retransplantation. Kaplan-Meier log-rank tests were used to test differences in patient survival, graft survival and death-censored graft failure between groups. The hazard ratios (HRs) and 95% confidence intervals (CIs) of overall graft failure, death-censored graft failure and death after KT were estimated using Cox proportional hazards model. A *p*-value less than 0.05 in a two-sided test was regarded as statistically significant. All statistical analyses were performed using SAS version 9.4 (SAS Institute, Cary, NC, USA) and R 3.6.3 (Vienna, Austria, http://www.R-project.org).

## Results

### Baseline characteristics of included KTs

**Fig 1:**
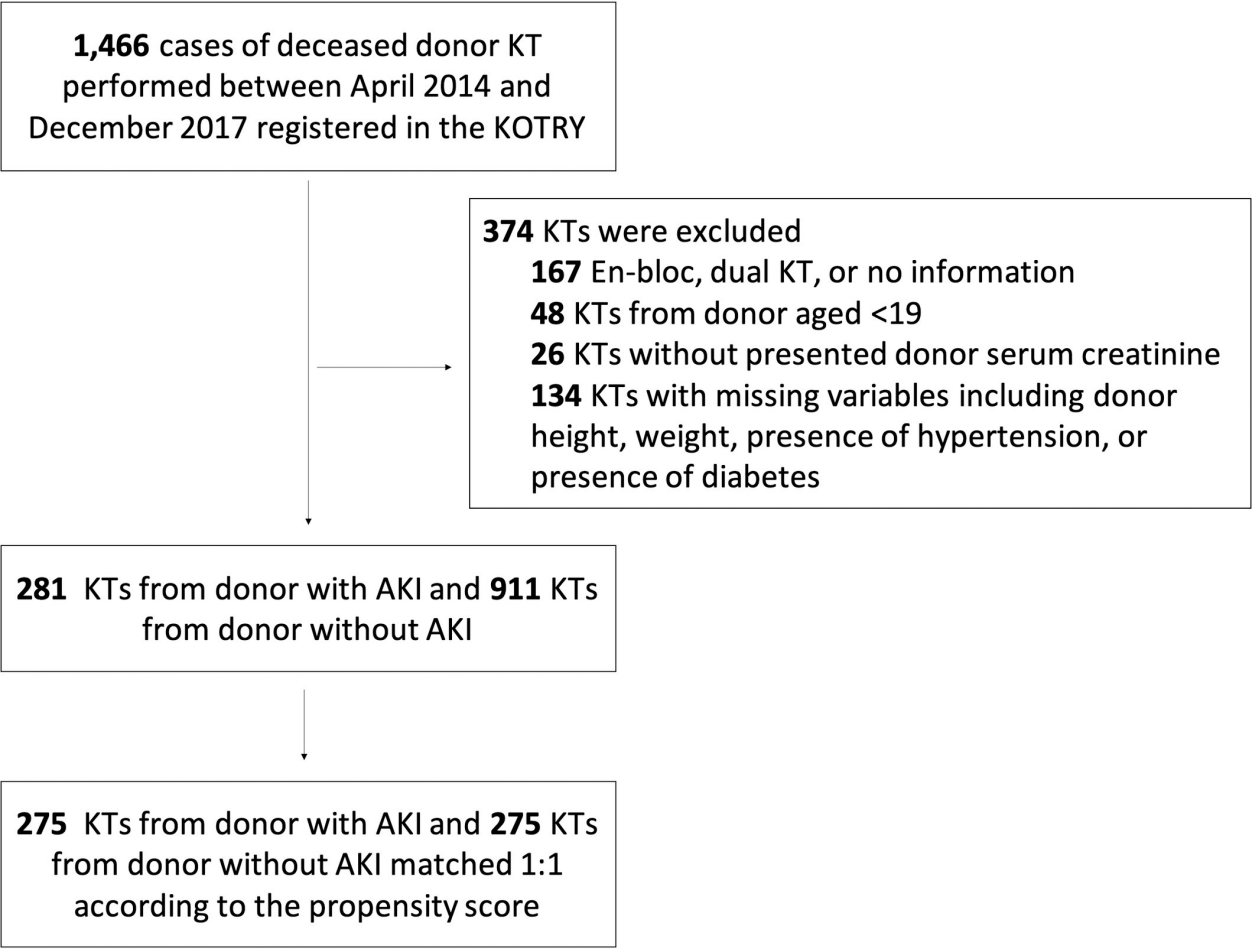
Flow diagram showing the selection of the study population.

Among 1,466 deceased donor KTs performed between April 2014 and December 2017 and identified from the KOTRY database, 275 KTs from donors with AKI were matched at 1:1 to 275 from donors without AKI according to the propensity score. Fig 1 provides information of excluded cases in detail. Characteristics of matched variables including SMDs by donor AKI status before and after matching are presented in S1 Table.

Baseline characteristics of donors and recipients included in the analysis and transplantation-related characteristics are described in Table 1 according to group. The mean age of donors was 46.7±13.4 years. The majority (83.6%) of donors were males. Their mean BMI was 23.9 ± 3.5 kg/m^2^. Of all donors, 42.6% died from cerebrovascular causes. Recipients were 51.1 ± 10.7 years old in average. Most (60.9%) of them were males. Their mean BMI was 23.1 ± 3.4 kg/m^2^. There were three leading primary causes of renal disease: glomerular disease (35.4%), diabetes (30.7%), and hypertension (28.6%). Mean serum creatinine levels of AKI donors and non-AKI donors were 3.55 ± 1.44 mg/dL and 1.05 ± 0.44 mg/dL (*p* < 0.001), respectively. Compared to non-AKI donors, AKI donors had higher mean KDRI (1.66 ± 0.40 versus 1.42 ± 0.38, *p* < 0.001) and KDPI (75 ± 18% versus 61 ± 22%, *p* < 0.001). Anti-thymocyte globulin was more frequently used as an induction agent in KTs from AKI donors than in KTs from non-AKI donors (44.7% versus 24.0%, *p* < 0.001). No significant difference in the frequency of patients using each maintenance immunosuppressant was observed.

**Table 1:** Baseline characteristics of subjects in KT from AKI donor group and KT from non-AKI donor groups.

	KT from AKI donor (n = 275)	KT from non-AKI donor (n = 275)	*P*-value
**Donor characteristics**			
Age, years	47.17 ± 12.11	46.29 ± 14.58	0.443
Male, No. (%)	227 (82.5)	233 (84.7)	0.564
Height, cm	169.48 ± 8.86	170.20 ± 8.11	0.323
Weight, kg	69.40 ± 11.75	68.83 ± 12.35	0.577
BMI, kg/m^2^	24.10 ± 3.48	23.66 ± 3.52	0.147
Diabetes, No. (%)	45 (16.4)	44 (16.0)	1.000
Hypertension, No. (%)	79 (28.7)	73 (26.5)	0.634
Serum creatinine, mg/dL	3.55 ± 1.44	1.05 ± 0.44	<0.001
Cerebrovascular death, No. (%)	119 (43.3)	115 (41.8)	0.796
Current or ex-smoker, No. (%)	145 (55.6)	127 (50.2)	0.259
ECMO, No. (%)	14 (5.1)	9 (3.3)	0.394
KDRI	1.66 ± 0.40	1.42 ± 0.38	<0.001
KDPI, %	75 ± 18	61 ± 22	<0.001
**Recipient characteristics**			
Age, years	51.83 ± 10.45	50.36 ± 10.72	0.106
Male, No. (%)	172 (62.5)	163 (59.3)	0.484
Height, cm	165.79 ± 8.25	164.65 ± 8.65	0.114
Weight, kg	63.12 ± 11.16	63.21 ± 12.23	0.935
BMI, kg/m^2^	22.88 ± 3.14	23.22 ± 3.57	0.235
Diabetes, No. (%)	82 (29.7)	80 (29.1)	0.925
Hypertension, No. (%)	254 (92.7)	243 (88.4)	0.112
Current or ex-smoker, No. (%)	78 (28.6)	59 (21.5)	0.072
Past history of KT, No. (%)	22 (8.0)	20 (7.27)	0.748
Duration of dialysis before KT, years	7.45 ± 4.90	7.54 ± 4.74	0.832
Primary cause of renal disease, No. (%)			
Diabetes	71 (32.0)	62 (29.4)	P for trend 0.244
Hypertension	69 (31.1)	55 (26.1)	
Glomerular disease	70 (31.5)	83 (39.3)	
Tubulointerstitial disease	0 (0.0)	2 (0.9)	
Polycystic kidney disease	12 (5.4)	9 (4.3)	
Other or unknown	53 (19.3)	64 (23.3)	
**Transplantation characteristics**			
CIT, hours	4.90 ± 2.22	4.97 ± 2.09	0.748
HLA mismatch score, out of 6	2.29 ± 1.63	2.47 ± 1.71	0.202
Baseline DSA positive, No. (%)	18 (11.3)	26 (15.3)	0.370
Induction medication, No. (%)			
Anti-thymocyte globulin	123 (44.7)	66 (24.0)	<0.001
Basiliximab	152 (55.3)	209 (76.0)	<0.001
Maintenance immunosuppressants, No. (%)			
Tacrolimus	270 (98.2)	273 (99.3)	0.450
Cyclosporin	4 (1.5)	0 (0)	0.124
Mycophenolic acid	262 (95.3)	258 (93.8)	0.453
Steroid	272 (98.9)	268 (97.5)	0.202

KT, kidney transplantation; AKI, acute kidney injury; SMD, standardized mean difference; BMI, body mass index; ECMO, extracorporeal membrane oxygenation; KDPI, kidney donor profile index; KDRI, kidney donor risk index; CIT, cold ischemic time; HLA, human leukocyte antigen; DSA, donor-specific antibody.

### Allograft outcomes

**Fig 2:**
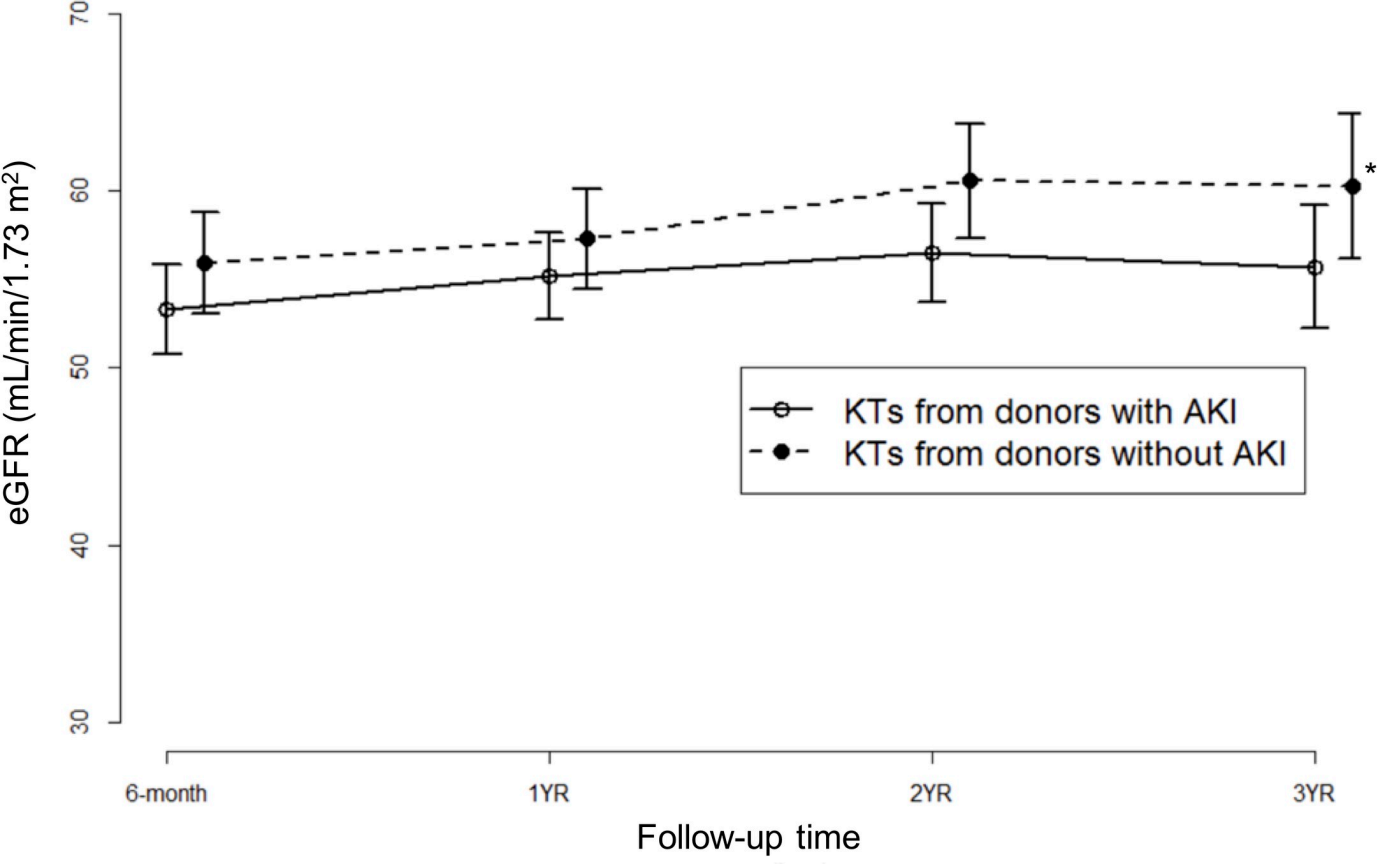
Mean eGFR in KT from AKI donors and non-AKI donors. eGFR, estimated glomerular filtration rate; KT, kidney transplantation; AKI, acute kidney injury. Generalized estimating equation showed significant difference in eGFR by time (*p* = 0.006). However, group versus time interaction was insignificant (*p* = 0.427). eGFR showed a significant difference (*, *p* < 0.05) at 6 months and 3 years after KT.

Allograft outcomes according to donor AKI status are presented in Table 2. The incidence of DGF was significantly higher in the group of KTs from AKI donors than in the group of KTs from non-AKI donors (24.5% versus 6.2%, *p* < 0.001). However, the incidence of PNF was not significantly different between the two groups (*p* = 0.157). There was a significant difference in eGFR by time (*p* = 0.006, Fig 2), although the variance in eGFR was not significantly different between KTs from AKI and non-AKI donors (*p* = 0.427). After KTs from non-AKI donors, eGFR was improved at 3 years compared to that at 6 months after KT (*p* = 0.002). There was no significant difference in the incidence of biopsy-confirmed rejection within 1 year or biopsy-confirmed BK nephropathy.

**Table 2:** Graft outcomes by AKI status of deceased donor.

Variables	KT from AKI donor	KT from non-AKI donor	P-value
eGFR, mL/min/1.73 m^2^			
6 months post-KT	53.29 ± 20.60	55.94 ± 23.44	0.172
1 year post-KT	55.20 ± 19.93	57.28 ± 22.69	0.279
2 years post-KT	56.53 ± 20.20	60.56 ± 22.80	0.064
3 years post-KT	55.71 ± 19.32	60.25 ± 22.19	0.099
PNF, No. (%)	2 (0.73)	0 (0.00)	0.157
DGF, No. (%)	67 (24.54)	17 (6.18)	<0.001
Biopsy-confirmed rejection within 1 year, No. (%)	43 (15.64)	54 (19.64)	0.2184
Borderline rejection within 1 year	17 (9.71)	20 (7.27)	0.7031
Acute T cell-mediated rejection	16 (5.82)	24 (8.73)	
Acute antibody-mediated rejection	5 (1.82)	7 (2.55)	
Mixed acute rejection	5 (1.82)	3 (1.09)	
Biopsy-confirmed BK nephropathy, No. (%)	3 (1.09)	6 (2.18)	0.504

KT, kidney transplantation; AKI, acute kidney injury; eGFR, estimated glomerular filtration rate; DGF, delayed graft function; PNF, primary nonfunction

### Allograft and patient survival

**Fig 3:**
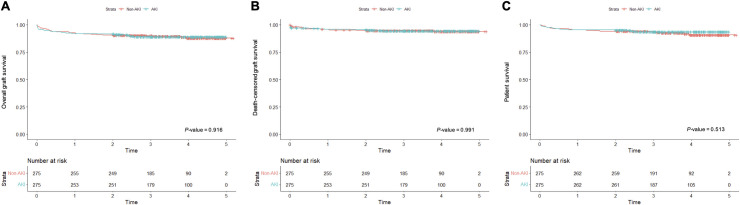
Overall graft survival (A), death-censored graft survival (B) and patient survival (C) after KT from donor with AKI and without AKI.

The median follow-up duration of recipients was 3.52 (interquartile range, 2.69–4.28) years. Graft and patient survivals by donor AKI status are shown in Fig 3. The risk of overall graft failure (HR, 0.973; 95% CI, 0.584 to 1.621), death-censored graft failure (HR, 1.004; 95% CI, 0.491 to 2.054) and mortality (HR, 0.808; 95% CI, 0.426 to 1.532) were not significantly different by donor AKI status.

## Discussion

The discard rate of deceased kidneys is increasing without showing significant difference in the quality between transplanted and discarded kidneys [21], implying that better assessment and distribution of such limited resource could increase the donor pool. Given that AKI donor is one of common causes of kidney discard, the impact of donor AKI status on outcomes of KT comes to the fore. Although preceding studies have reported favorable outcomes of KTs from AKI donors [4–8, 10, 12–14, 17, 18] and feasible mechanisms have been proposed [11, 22], there have been considerable discordances in the selection criteria for kidneys from AKI donors across centers [7, 12]. Due to low feasibility of randomized controlled trials, well-designed observational studies with low risk of bias and confounders could provide decisive evidence.

To the best of our knowledge, this is the first nationwide cohort study reporting serial graft function after KTs according to donor AKI status in an Asian population. We found that KTs from AKI donors resulted in comparable outcomes to propensity score-matched KTs from non-AKI donors in an Asian population. According to our findings, despite higher incidence of DGF in KTs from AKI donors, the rejection rate, eGFR, graft survival, and patient survival were not significantly affected by donor AKI status. These findings are in line with prior studies including one study on 6,722 deceased donors with AKI matched to donors without AKI showing that AKI status of donor can significantly increase the risk of DGF (29% versus 22%, p < 0.001), while AKI status was not related to death-censored graft failure (HR: 1.01; 95% CI: 0.95 to 1.08) or all-cause graft failure (HR: 0.97; 95% CI: 0.93 to 1.02) [11]. Higher incidence of DGF relevant to donor AKI has been consistently featured in abundant studies. However, convincing short-term and long-term outcomes justify the use of AKI kidneys [23].

Our study population composed of Asian with short CIT (mean, 4.94 ± 2.16 hours), one of the most important factors affecting graft survival and function [24, 25]. In addition, basiliximab was predominantly prescribed as an induction agent attributing to a low immunologic risk of Asian [26]. Before cases were matched by propensity score, the study cohort showed that AKI donors tended to be taller and heavier than non-AKI donors (S1 Table). This tendency can be explained by choosing favorable characteristics offsetting higher risk of poor prognosis after KT. The size of kidney, which is proportional to the body size of the donor, is related to better prognosis after KT [27, 28].

We observed a significant increase in kidney function measured at 3 years compared to 6 months after KTs from non-AKI donors (Fig 2), which was also reported in recent studies [29, 30]. Although the exact mechanism has not been elucidated yet and its clinical significance is controversial, several plausible explanations could be suggested. First, improved kidney function over time could be explained as a compensatory hyperfiltration of the graft in a fashion similar to improvement of renal function in live donors after nephrectomy [31]. Second, improvements in general medical care for transplant patients and management for high immunologic risk patients and acute rejection episodes with better choice of immune suppression [32, 33] might have contributed to better graft functions after KT. Third, increase in eGFR within 3 years after KTs could be explained as a natural course of recovery from pre-, intra-, and post-transplant graft injury such as ischemic insults during the donor/recipient management and ischemia reperfusion injury which is an inevitable consequence following KTs [33].

In this study, there was no significant difference in the mean eGFR measured at each follow-up period between KTs from AKI and KTs from non-AKI donors. Previous long-term observation studies with multiple eGFR measurements to compare graft functions between KTs from AKI and non-AKI donors have shown inconsistent results. Some studies showed that eGFR after KTs from AKI donors was comparable to that after KTs from non-AKI donors until 5 years [12, 17]. In contrast, Bauer et al. [18] found that KTs from AKI and non-AKI donors did not show significant difference in eGFR measured at 1 year after KT; however, eGFR at 3 and 5 years after KT was significantly higher in the non-AKI group than in the AKI group. Schütte-Nütgen et al. [34] also reported that KTs from AKI donors consistently show lower eGFR than KTs from non-AKI donors at 3-year follow-up.

Some limitations of this study should be acknowledged. First, we used dichotomized definition of AKI donors without considering chronicity, severity, or dynamic changes in donor kidney function as donor serum creatinine level was reported only once in the registry. Second, although bias from the retrospective design of this study was partially resolved by propensity score matching, there might be effects of known and unknown variables. Third, a considerable number of cases were excluded due to missing variables. Fourth, the small cohort size decreased the statistical power of this study.

Taken together, this study supports the validity of using kidneys from deceased AKI donors in Asian population. Further studies with long term follow-up duration are needed to investigate graft function according to donor AKI status.

## Supporting information

S1 Table Characteristics of matched variables according to donor AKI status before and after propensity score matching. (DOCX)

## References

[1] Purnell TS, Auguste P, Crews DC, et al. Comparison of life participation activities among adults treated by hemodialysis, peritoneal dialysis, and kidney transplantation: a systematic review. *American Journal of Kidney Diseases*. 2013;62:953–973. doi: 10.1053/j.ajkd.2013.03.022 23725972 PMC3809150

[2] Schold JD, Buccini LD, Goldfarb DA, Flechner SM, Poggio ED, Sehgal AR. Association between kidney transplant center performance and the survival benefit of transplantation versus dialysis. *Clinical Journal of the American Society of Nephrology*. 2014;9:1773–1780. doi: 10.2215/CJN.02380314 25237071 PMC4186511

[3] Port FK, Bragg-Gresham JL, Metzger RA, et al. Donor characteristics associated with reduced graft survival: an approach to expanding the pool of kidney donors. *Transplantation*. 2002;74:1281–1286. doi: 10.1097/00007890-200211150-00014 12451266

[4] Hall IE, Schröppel B, Doshi MD, et al. Associations of deceased donor kidney injury with kidney discard and function after transplantation. *American Journal of Transplantation*. 2015;15:1623–1631. doi: 10.1111/ajt.13144 25762442 PMC4563988

[5] Kayler L, Garzon P, Magliocca J, et al. Outcomes and utilization of kidneys from deceased donors with acute kidney injury. *American Journal of Transplantation*. 2009;9:367–373. SRTR doi: 10.1111/j.1600-6143.2008.02505.x 19178415

[6] Hall IE, Akalin E, Bromberg JS, et al. Deceased-donor acute kidney injury is not associated with kidney allograft failure. *Kidney international*. 2019;95:199–209. multicenter doi: 10.1016/j.kint.2018.08.047 30470437 PMC6331055

[7] Heilman RL, Smith ML, Smith BH, et al. Long-term outcomes following kidney transplantation from donors with acute kidney injury. *Transplantation*. 2019;103:e263–e272. Single center doi: 10.1097/TP.0000000000002792 31205261

[8] Kumar MSA, Khan SM, Jaglan S, et al. Successful transplantation of kidneys from deceased donors with acute renal failure: three-year results. *Transplantation*. 2006;82:1640–1645. doi: 10.1097/01.tp.0000250908.62948.8f 17198251

[9] Kim JH, Kim YS, Choi MS, et al. Prediction of clinical outcomes after kidney transplantation from deceased donors with acute kidney injury: a comparison of the KDIGO and AKIN criteria. *BMC nephrology*. 2017;18:39. 강남, 의정부 성모 doi: 10.1186/s12882-017-0461-5 28129763 PMC5273789

[10] Klein R, Galante NZ, de Sandes-Freitas TV, de Franco MF, Tedesco-Silva H, Medina-Pestana JO. Transplantation with kidneys retrieved from deceased donors with acute renal failure. *Transplantation*. 2013;95:611–616. doi: 10.1097/TP.0b013e318279153c 23274968

[11] Liu C, Hall IE, Mansour S, Philbrook HRT, Jia Y, Parikh CR. Association of deceased donor acute kidney injury with recipient graft survival. *JAMA Network Open*. 2020;3:e1918634. doi: 10.1001/jamanetworkopen.2019.18634 31913491 PMC6991314

[12] Jadloweic CC, Heilman RL, Smith ML, et al. Transplanting kidneys from donation after cardiac death donors with acute kidney injury. American Journal of Transpalntation. 2020;20:864–869. Single center 10.1111/ajt.15653 31612611

[13] Boffa C, Van de Leemkolk F, Curnow E, et al. Transplantation of kidneys from donors with acute kidney injury: friend or foe? *American Journal of Transplantation*. 2017;17:411–419. doi: 10.1111/ajt.13966 27428556

[14] Jung CW, Park K, Kim S, et al. Clinical outcomes in kidney transplantation patients from deceased donors with acute kidney injury. *Transplantation proceedings*. Elsevier; 2013: 2941–2945. Single center, short term doi: 10.1016/j.transproceed.2013.08.048 24157008

[15] Heilman R, Smith M, Kurian S, et al. Transplanting kidneys from deceased donors with severe acute kidney injury. *American Journal of Transplantation*. 2015;15:2143–2151. Single center doi: 10.1111/ajt.13260 25808278

[16] Farney AC, Rogers J, Orlando G, et al. Evolving experience using kidneys from deceased donors with terminal acute kidney injury. *Journal of the American College of Surgeons*. 2013;216:645–655. doi: 10.1016/j.jamcollsurg.2012.12.020 23395159

[17] Domagala P, Gorski L, Wszola M, et al. Successful transplantation of kidneys from deceased donors with terminal acute kidney injury. *Renal failure*. 2019;41:167–174. doi: 10.1080/0886022X.2019.1590209 30909784 PMC6442227

[18] Bauer J, Grzella S, Bialobrzecka M, et al. Success of kidney transplantations from deceased donors with acute kidney injury. *Annals of transplantation*. 2018;23:836. doi: 10.12659/AOT.912660 30523243 PMC6298175

[19] Rao PS, Schaubel DE, Guidinger MK, et al. A comprehensive risk quantification score for deceased donor kidneys: the kidney donor risk index. *Transplantation*. 2009;88:231–236. doi: 10.1097/TP.0b013e3181ac620b 19623019

[20] Stevens LA, Coresh J, Greene T, Levey AS. Assessing kidney function—measured and estimated glomerular filtration rate. *New England Journal of Medicine*. 2006;354:2473–2483. doi: 10.1056/NEJMra054415 16760447

[21] Mohan S, Chiles MC, Patzer RE, et al. Factors leading to the discard of deceased donor kidneys in the United States. *Kidney international*. 2018;94:187–198. doi: 10.1016/j.kint.2018.02.016 29735310 PMC6015528

[22] Puthumana J, Hall IE, Reese PP, et al. YKL-40 associates with renal recovery in deceased donor kidney transplantation. *Journal of the American Society of Nephrology*. 2017;28:661–670. doi: 10.1681/ASN.2016010091 27451287 PMC5280016

[23] Chan GCK, Chow KM. Should we use kidneys from donors with acute kidney injury for renal transplantation? *Nephrology*. 2020;25:105–115. doi: 10.1111/nep.13679 31707757

[24] Summers DM, Johnson RJ, Allen J, et al. Analysis of factors that affect outcome after transplantation of kidneys donated after cardiac death in the UK: a cohort study. *The Lancet*. 2010;376:1303–1311. doi: 10.1016/S0140-6736(10)60827-6 20727576

[25] Kayler L, Magliocca J, Zendejas I, Srinivas T, Schold J. Impact of cold ischemia time on graft survival among ECD transplant recipients: a paired kidney analysis. *American journal of transplantation*. 2011;11:2647–2656. doi: 10.1111/j.1600-6143.2011.03741.x 21906257

[26] Chang J-Y, Yu J, Chung BH, et al. Immunosuppressant prescription pattern and trend in kidney transplantation: A multicenter study in Korea. *PloS one*. 2017;12:e0183826. doi: 10.1371/journal.pone.0183826 28846737 PMC5573298

[27] Miles AMV, Sumrani N, John S, et al. The effect of kidney size on cadaveric renal allograft outcome. *Transplantation*. 1996;61:894–897. doi: 10.1097/00007890-199603270-00009 8623156

[28] Moreso F, Serón D, Anunciada AI, et al. Recipient body surface area as a predictor of posttransplant renal allograft evolution. *Transplantation*. 1998;65:671–676. doi: 10.1097/00007890-199803150-00012 9521202

[29] Gourishankar S, Hunsicker LG, Jhangri GS, Cockfield SM, Halloran PF. The stability of the glomerular filtration rate after renal transplantation is improving. *Journal of the American Society of Nephrology*. 2003;14:2387–2394. doi: 10.1097/01.asn.0000085019.95339.f0 12937318

[30] Srinivas TR, Flechner SM, Poggio ED, et al. Glomerular filtration rate slopes have significantly improved among renal transplants in the United States. *Transplantation*. 2010;90:1499–1505. doi: 10.1097/TP.0b013e3182003dda 21085061

[31] Bertolatus JA, Friedlander MA, Scheidt C, Hunsicker LG. Urinary albumin excretion after donor nephrectomy. *American Journal of Kidney Diseases*. 1985;5:165–169. doi: 10.1016/s0272-6386(85)80045-7 3883759

[32] Lim MA, Kohli J, Bloom RD. Immunosuppression for transplantation: Where are we now and where are we going? *Transplantation Reviews*. 2017;31:10–17. doi: 10.1016/j.trre.2016.10.006 28340885

[33] Salvadori M, Rosso G, Bertoni E. Update on ischemia-reperfusion injury in kidney transplantation: Pathogenesis and treatment. *World Journal of Transplantation*. 2015;5:52–67. doi: 10.5500/wjt.v5.i2.52 26131407 PMC4478600

[34] Schütte-Nütgen K, Finke M, Ehlert S, et al. Expanding the donor pool in kidney transplantation: Should organs with acute kidney injury be accepted?—A retrospective study. *PloS one*. 2019;14:e0213608. doi: 10.1371/journal.pone.0213608 30865677 PMC6415810

