# Supplementary material for: Impact of acute kidney injury on graft outcomes of deceased donor kidney transplantation: A nationwide registry-based matched cohort study in Korea
Source: PLoS One. 2021 Nov 17;16(11):e0260076. doi: 10.1371/journal.pone.0260076 (PMC8598029; doi:10.1371/journal.pone.0260076)
Supplement: S1 Table — (DOCX) [file pone.0260076.s001.docx]

| **S1 Table. Characteristics of matched variables according to donor AKI status before and after propensity score matching** | | | | | | | |
| --- | --- | --- | --- | --- | --- | --- | --- |
| **Donor characteristics** | **Before matching** | | |  | **After matching**  **(Study population)** | | |
|  | KT from AKI donor (n=304) | KT from non-AKI donor (n=969) | SMD |  | KT from AKI donor (n= 275) | KT from non-AKI donor (n= 275) | SMD |
| Age, years | 47.50 ±11.95 | 48.14 ± 15.35 | 0.047 |  | 47.17 ± 12.11 | 46.29 ± 14.58 | 0.065 |
| Male, No. (%) | 249 (81.9) | 640 (66.0) | 0.368 |  | 227 (82.5) | 233 (84.7) | 0.059 |
| Height, cm | 169.25 ±8.78 | 165.79 ±12.30 | 0.323^*^ |  | 169.48 ± 8.86 | 170.20 ± 8.11 | 0.084 |
| Weight, kg | 69.87 ±12.44 | 63.42 ±13.45 | 0.498^*^ |  | 69.40 ± 11.75 | 68.83 ± 12.35 | 0.048 |
| Diabetes, No. (%) | 45 (15.6) | 123 (13.1) | 0.070 |  | 45 (16.4) | 44 (16.0) | 0.010 |
| Hypertension, No. (%) | 87 (30.1) | 236 (25.5) | 0.104 |  | 79 (28.7) | 73 (26.5) | 0.049 |
| Cerebrovascular death, No. (%) | 138 (45.4) | 403 (41.6) | 0.077 |  | 119 (43.3) | 115 (41.8) | 0.029 |
| Abbreviations: KT, kidney transplantation; AKI, acute kidney injury; SMD, standardized mean difference.  ^*^ Difference between groups with statistical significance at *p-*value < 0.05. | | | | | | | |
